# Surgical Treatment of Sub-Acute Nephritis

**Published:** 1920-12-04

**Authors:** 


### SURGICAL TREATMENT OF SUB-ACUTE NEPHRITIS.

Within the last few weeks Sir Thomas Horder, M.D., F.R.C.P., has read a most interesting paper on this subject before the Urological Section of the Royal Society of Medicine, and the following is a brief extract of his remarks. During the decade 1896-1906 a good deal of experimental surgery was undertaken in connection with certain inflammatory conditions of the kidney which had not formerly been considered amenable to such methods. The treatment was at first associated with the names of Harrison, Edebohls, Israel, and Poussen; and later those of Boyd, Tyson, and Clayton-Greene.

The Early Technique.

The technique adopted was variable, the earliest method being a simple puncture of the kidney capsule; this was followed by simple incision: both of these methods were used in cases of acute inflammation, either when the kidney was considered to be in a state of tension within its capsule, or when the patient was anuric. Still later, the capsule was not only incised, but more or less of it was stripped from the kidney. In the earlier procedures the kidney wound was allowed to ooze " blood and urine " through a drainage tube for several days; in the later operations the kidney was returned to its bed after the capsule had been stripped, and the wound was sutured forthwith. In the earlier cases, again, one kidney only was dealt with, or at first one, and then, if it seemed desirable, the other kidney was similarly treated some days afterwards; whilst in the later instances both kidneys were subjected to decapsulation at one and the same operation.

The Present Cases.

Of the four cases of decapsulation forming the basis of the present paper the first was undertaken in 1914 and the last in 1919. The patients varied in age between ten and thirty years, and in each case there was well marked clinical evidence of nephritis, such as œdema of the limbs and face, ascitis, headaches, lack of energy and nausea; chemical analysis of the urines also revealed the pathological contents associated with this disease.

In all four cases both kidneys were decapsulated at the same operation, the capsules being completely stripped from the entire organs.

The operation is quite simple in the hands of an expert surgeon, and complete decapsulation of both kidneys can generally be completed in about half-an-hour. In each of these cases more or less complete recovery was observed.

Conclusions.

Although—as Sir Thomas Horder wisely remarks—it would be absurd to dogmatise upon so little material, yet consideration of these four cases, together with careful analysis of all the available records, leads to the conclusion that there is a clinical type of nephritis in which—when general measures have proved unavailing—decapsulation becomes a definite indication and promises satisfactory results.

This type is not inappropriately termed *sub-acute nephritis*, and is characterised by extensive and considerable œdema, massive albuminuria with casts in the urine, toxic symptoms of the chronic uræmic kind, and an absence, or the presence in only slight degree, of cardio vascular changes.

Concerning the mechanism by which the results of decapsulation are brought about, there is little known, but it is generally thought that depriving a kidney of its capsule, and thus bringing the contex into close contact with the perinephric tissues, leads to the formation of new vessels which anastomose with those of the kidney itself, thus providing for the removal of inflammatory exudates and assisting in re-establishing renal function. Against this theory, we have the clinical fact, that relief *immediately* follows decapsulation, and long before these new vessels would have had time to develop: thus, the question for a time must remain unanswered—unless it be an answer to say that the mechanism of production of the diaresis and disappearance of œdema and of symptoms generally is of the same kind as in the process of " wet cupping."

The whole subject calls for systematic study, but already a new avenue of hope would seem to open to sufferers from nephritis in general, and sub-acute nephritis in particular.

